# THE PIPELINE OF GEROPSYCHOLOGY: INTEGRATION OF CLINICAL AND ACADEMIC CAREER TRAJECTORIES

**DOI:** 10.1093/geroni/igz038.036

**Published:** 2019-11-08

**Authors:** Hillary Dorman, Jessica V Strong, Caitlan A Tighe, Benjamin T Mast, Rebecca S Allen

**Affiliations:** 1 University of Alabama, Tuscaloosa, Tuscaloosa, Alabama, United States; 2 VA Boston Healthcare System, Brockton, Massachusetts, United States; 3 VA Pittsburgh Healthcare System, Pittsburgh, Pennsylvania, United States; 4 University of Louisville, Louisville, Kentucky, United States; 5 The University of Alabama, Tuscaloosa, Alabama, United States

## Abstract

The current exploratory study aimed to identify factors influencing gerospychologists’ career trajectories, discerning what is “attractive” and “unattractive” across common career options. Participants rated career characteristics similarly (higher starting salary attractive, administrative tasks unattractive in a clinical VA; setting own hours attractive, few collaboration opportunities unattractive in a clinical non-VA; work variety attractive, publication expectations unattractive in academia). Interestingly, men found a clinical VA’s set hours more unattractive (t(50) = -2.59, p = .010). Sex differences also were evident in career change feasibility. 50% of respondents would consider switching careers (61% clinical to academia, 79% academia to clinical). Men were significantly more likely to consider switching from clinical to academia (χ2(2) = 6.33, p = .042) and find it feasible (χ2(2) = 8.92, p = .012). Enhancing our understanding of what encourages/discourages careers in geropsychology is needed to address the workforce shortage; this study takes important steps in this direction.

